# Using Integrins for Tumor Imaging

**DOI:** 10.1371/journal.pmed.0020091

**Published:** 2005-03-29

What holds cells together or connects them with the extracellular matrix—and what happens when these interactions break down—is one of the keys to determining how tumors metastasize. One group of compounds—integrins—are a central part of these interactions. Not only do integrins play a part in cell–cell and cell–matrix adhesion, but they also are involved in signal transduction (the method by which a cell relays information from receptor binding to cellular response) and in triggering cell death by linking to other molecules. One such member of this receptor family is the avß3 integrin, which is expressed on both the tumor cells and the new vasculature of various tumors, including melanomas. avß3 integrin has a role in cell migration and extravasation, which occurs during metastasis, and also in angiogenesis—the development of new blood vessels that are essential for the growth of tumors. These blood vessels are the target for one class of anti-cancer drugs—angiogenesis inhibitors. Molecules that bind to avß3 integrin have also been used to target therapeutic compounds to tumors: compounds that antagonize this integrin can lead to apoptosis (programmed cell death) of cells that express it.

Haubner and colleagues, the authors of a paper in this month's *PLoS Medicine*, have previously developed a fluorine-labeled peptide, [^18^F]Galacto-RGD, that has a high affinity for avß3 integrin. [^18^F]Galacto-RGD has many of the features essential for a tracer: it is specifically accumulated by tumors that express avß3 integrin, it is efficiently eliminated by the kidneys, and it is stable in vitro and in vivo.

In the research paper in *PLoS Medicine*, Haubner and colleagues take the development of the compound further towards clinical application. First, in a mouse with human melanoma they used highly sensitive positron emission tomography (PET) scanning to show not only that the level of uptake of integrin was specific for the tumor, but also that the uptake was in direct proportion to the amount of avß3 expressed, thus potentially allowing quantification of receptor expression; however, larger tumors showed a poorer correlation, possibly because of the presence of necrotic areas that do not express the integrin.

In humans, this picture was a little less clear; in a small study of patients with tumors including melanoma, the authors found a good deal of difference between patients in the uptake of the marker by tumor cells and the corresponding tumor vasculature. However, there was good correlation between the tracer uptake and conventional staining for the integrin by immunohistochemistry—again suggesting that the marker is truly reflecting the in vivo level of the integrin.

What do these results mean for clinical applications? As well as identifying tumors that express this marker, this approach might also offer a noninvasive way to assess the degree of new vessel formation in tumors. The approach could provide important information for planning and monitoring anti-angiogenic therapies targeting this integrin and could reveal the involvement and role of this integrin in metastatic and angiogenic processes in various diseases.

**Figure:**
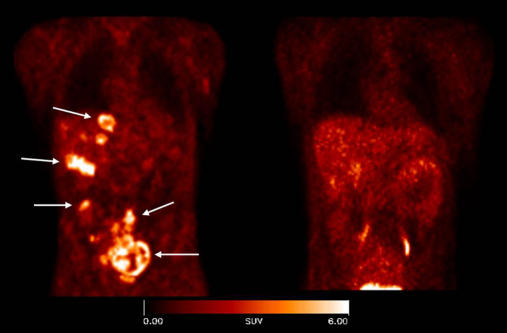
avß3 integrin expression in tumors

